# Perspectives and Experiences of Nurse Managers on the Impact of Artificial Intelligence on Nursing Work Environments and Managerial Processes: A Qualitative Study

**DOI:** 10.1111/inr.70043

**Published:** 2025-06-18

**Authors:** Nilgün Göktepe, Seda Sarıköse

**Affiliations:** ^1^ Department of Nursing Ordu University Faculty of Health Science Ordu Türkiye; ^2^ Koç University School of Nursing Istanbul Türkiye

**Keywords:** artificial intelligence, nurse managers, nursing, qualitative research, work environment

## Abstract

**Aim:**

To explore the perspectives and experiences of nurse managers regarding the impact of artificial intelligence (AI) on nursing work environments and managerial processes.

**Background:**

The integration of AI into nursing care delivery has emerged as a critical component of the evolving role of nurse managers. AI‐supported technologies provide nurse managers with advanced tools to facilitate patient monitoring, predict high hospital bed occupancy rates, and develop workforce planning strategies.

**Methods:**

This study used a descriptive design with semi‐structured interviews to explore the experiences of 22 nurse managers. Qualitative data were analyzed through content analysis. The Consolidated Criteria for Reporting Qualitative Research have been followed by reporting the methods and findings.

**Results:**

This study identified four main themes regarding nurse managers’ perspectives on the impact of AI in nursing: applications of AI, reflections on the nursing environment, barriers to use, and strategies for integration, along with 12 related subthemes.

**Conclusions:**

This study highlights both the opportunities and challenges of integrating AI into nursing practices and management. While AI can enhance patient safety, care quality, and resource management, barriers such as infrastructure deficiencies, data security concerns, and cultural resistance hinder its implementation.

**Implications for nursing and nursing policy:**

The findings suggest that AI can be a strategic tool in nursing work environment and clinical practices, shaping nursing leadership and health policies. Organizational readiness, support, and empowering nurse managers through AI leadership emerge as critical drivers for successful integration.

## 1 Introduction

As in all professional fields, information and communication technologies are rapidly advancing, significantly impacting the nursing profession (Booth et al. 2021). In the future, nurses will be able to integrate their knowledge and skills with artificial intelligence (AI)‐supported technologies, enabling them to perform their roles in healthcare more effectively and efficiently (Oksanen et al. 2020). AI is an advanced technological replication of human intelligence. It also incorporates systems capable of making logical and mathematical decisions (Zhao et al. 2022). With their analytical and learning capabilities, AI‐supported technologies can execute processes faster, more efficiently, and at a lower cost (Oksanen et al. 2020). AI and robotic technologies are becoming indispensable in healthcare systems (Chang et al. 2021). The use of AI has a multifaceted impact on nursing work environments, patient care, and administrative processes (Frazier et al. 2019).

The use of AI‐supported technologies in healthcare delivery has been shown to have positive effects on patient outcomes, such as reducing mortality rates, enhancing patient safety, and shortening hospital stays (Oksanen et al. 2020; Chang et al. 2022; Ng et al. 2022). Additionally, AI is expected to improve nurses’ adherence to evidence‐based nursing practices, support medication management and safety, and reduce risks such as falls, infections, and extubations through AI‐supported early warning systems (Chang et al. 2022; Choudhury and Asan 2020). According to the literature, increased workloads adversely affect nursing care practices and nurses’ quality of life (Khatatbeh et al. 2022). AI‐supported technologies facilitate nurses’ clinical decision‐making processes, allowing for greater efficiency and enabling them to play a more active role in patient care (Frazier et al. 2019).

Healthcare systems face numerous challenges, including rising costs, population growth, inequalities in access to services, and workforce shortages (Roncarolo et al. 2017). In addressing these challenges, using AI‐supported technologies in healthcare has gained prominence (Zhao et al. 2022). In Türkiye, the number of nurses per 100,000 people is 343. Nurses constitute 45% of all healthcare professionals, and the issue of insufficient staffing remains a priority concern (General Directorate of Health Information Systems 2021). Consequently, integrating AI‐supported patient care systems is recommended in nursing work environments due to their potential to optimize the efficient use of the nursing workforce (Ventura‐Silva et al. 2024). These technologies are viewed as supportive systems to mitigate the adverse effects of workforce shortages (Frazier et al. 2019). The contributions of AI‐supported technologies are expected to enhance efficiency and support understaffed healthcare environments.

Although research on AI in nursing has expanded significantly, there is limited focus on the role of nurse managers in AI implementation. Nurse managers play a critical role in overseeing nursing workflows, ensuring patient safety, and leading organizational change (Alsadaan et al. 2023). Their leadership is essential in determining how AI‐supported technologies are adopted, integrated, and accepted within healthcare institutions (Ross et al. 2024). Studies indicate that nurse managers who actively promote AI adoption can significantly improve the willingness of their teams to engage with these technologies, leading to increased efficiency and reduced resistance (Kotp et al. 2025; Ross et al. 2024). Similarly, a recent review highlights that nurse managers who provide ongoing education and support regarding AI applications foster a more positive perception of AI among nursing staff, leading to better utilization of AI‐driven tools in patient care (Gonzalez‐Garcia et al. 2024). Understanding their perspectives is key to identifying the facilitators and barriers to AI adoption in nursing environments.

## 2 Background

Historically, technologies such as electronic health records, mobile health, telehealth, and computer‐aided clinical decision support systems have been utilized to enhance nursing service quality and ensure safe patient care (Locsin 2017). The integration of AI into nursing care delivery is now regarded as one of the emerging roles of nurse managers (Ross et al. 2024). Nurse managers play a pivotal role in reconceptualizing nursing care services and work environments through the support of AI (Robert 2019). AI‐supported technologies provide nurse managers with advanced tools to facilitate patient monitoring, predict high hospital bed occupancy rates, and develop forward‐looking workforce planning strategies (Robert 2019; Ross et al. 2024). For instance, in high‐turnover units such as emergency departments, AI can calculate time‐dependent patient admissions, enabling the anticipation of workload for upcoming days (Zhao et al. 2022). From an administrative perspective, the use of AI is anticipated to reduce healthcare service costs and enhance service quality when nurses' expertise, knowledge, and critical thinking skills are combined with AI capabilities (Douthit et al. 2020).

Despite these positive effects, the widespread adoption of AI in nursing work environments faces significant challenges, primarily related to its integration into the profession (Ramadan et al. 2024; Hassan and El‐Ashry 2024). Nurse managers must lead efforts to encourage nurses to embrace the use of AI in care delivery (Hassan and El‐Ashry 2024). Some studies identify organizational resistance to sharing patient data, a shortage of qualified personnel, and the perception of AI as a threat to the workforce as key managerial barriers during the adaptation of AI technologies in healthcare institutions (Petersson et al. 2022; Ramadan et al. 2024). Additionally, AI‐supported technologies pose ethical dilemmas and disadvantages for nurses (Almagharbeh et al. 2025). Issues such as patient privacy, safety, and ethical concerns are among these challenges (Ramadan et al. 2024). Moreover, nurse managers' strategic decisions directly impact AI adoption within nursing teams (Ross et al. 2024). They are responsible for navigating ethical concerns, addressing workforce resistance, and ensuring that AI‐driven processes align with nursing values and patient‐centered care (Kotp et al. 2025; Mohammed et al. 2025). Research suggests that nursing units with strong managerial support for AI adoption experience higher job satisfaction among nurses and better patient outcomes (Chang et al. 2022). Therefore, exploring nurse managers’ perspectives is imperative in understanding how AI can be effectively implemented in nursing environments.

Nurse managers can facilitate the integration of AI into nursing work environments within the framework of Rogers’ diffusion of innovations (DOI) theory, which defines five key attributes influencing the adoption of innovations (Rogers 2003). In this context, the theoretical framework of this study builds on these five attributes to explore nurse managers’ perspectives and experiences of AI adoption in nursing work environments. Relative advantage refers to an innovation's economic and functional benefits, emphasizing how it enhances existing practices and outcomes. Compatibility denotes the degree to which an innovation aligns with current workflows, values, and needs, influencing how seamlessly it can be incorporated. Simplicity pertains to the ease of understanding and using innovation, as complex systems may create resistance, whereas user‐friendly solutions encourage adoption. Trialability refers to the possibility of testing an innovation before full implementation, allowing users to assess its benefits and feasibility in a controlled manner. Observability reflects the extent to which an innovation's positive effects are visible, fostering wider acceptance through demonstrable success (Rogers 2003). By utilizing these five key attributes in Rogers’ DOI theory, nurse managers play a crucial role in facilitating AI adoption in nursing, ensuring that innovations are effectively integrated, potential barriers are addressed, and nurses are supported in adapting to technological advancements. The strategic adoption of this framework in nursing work environments, where efficiency, patient safety, and workload management are key considerations, can help ensure a smoother transition to AI‐enabled practices. This approach may also enhance collaboration and improve decision‐making processes. Nurse managers can facilitate an evidence‐based approach to AI implementation by providing thoughtful guidance on digital transformation while maintaining alignment with professional and organizational goals.

## 3 Methods

### 3.1 Aim

This study aims to explore the perspectives and experiences of nurse managers regarding the impact of AI on nursing work environments and managerial processes.

### 3.2 Design

This study is designed as descriptive research utilizing the semi‐structured individual in‐depth interview method, one of the qualitative research approaches (Polit and Beck 2012). The study follows the Consolidated Criteria for Reporting Qualitative Research (COREQ) Checklist (Tong et al. 2007), which is widely recognized in the literature and recommended for the design and reporting of qualitative research (Supplemental File S1).

### 3.3 Participants

This study used the maximum variation sampling method, one of the purposive sampling methods, and was completed with a total of 22 nurse managers, 17 female and five male nurses who volunteered to participate in the study, working in first‐line, middle‐ and top‐level nurse manager positions in the Ministry of Health, university and private hospitals in a province in the western region of Türkiye. All participants were selected based on their willingness to share their experiences and insights with the researchers. To ensure maximum diversity, nurses with different educational levels (work and managerial experiences, genders, marital statuses, managerial positions, and institutions) were selected to participate in the study, and the characteristics of the participants are shown in Table 1. Data collection continued until data saturation was achieved, and all nurse managers participated voluntarily.

**TABLE 1:** Descriptive characteristics of the nurse manager.

Variables (*N* = 22)	Category	*n* (%)
Age (years)	Mean ± SD (min: 24; max: 56)	29.64 ± 4.45
Age group	<29	10 (45)
	≥29	12 (55)
Gender	Male	5 (22.73)
	Female	17 (77.27)
Educational level	Associate degree	3 (13.64)
	Bachelor's degree	15 (68.18)
	Postgraduate	4 (18.18)
Institution	State hospital	2 (9)
	Foundation/Private hospital	13 (59)
	University hospital	7 (32)
Position	First‐line nurse manager	9 (40.90)
	Midlevel nurse manager	6 (27.27)
	Executive/Top‐level nurse manager	7 (31.83)
Managerial experience (years)	Mean ± SD	7.78 ± 4.73
Managerial experience group	<7	10 (45)
	≥7	12 (55)

### 3.4 Research Team

The research team consisted of two researchers (female), with the first researcher holding a PhD degree in nursing management and the second researcher holding a PhD degree in nursing. Both researchers are faculty members in nursing. They have received prior training in qualitative research and have experience conducting such studies. Individual in‐depth interviews with participants were conducted by the second researcher (SS). Both researchers contributed to data analysis, theme development, and manuscript preparation. No formal and managerial relationship existed between the researchers and participants. At the beginning of the interviews, participants were provided with general information about the researchers.

### 3.5 Data Collection

In this study, two instruments were utilized: a participant information form consisting of six questions designed to collect descriptive information on participants' individual and socio‐demographic characteristics, and a semi‐structured interview form developed by the researchers, containing ten questions. The semi‐structured interview form was reviewed by three experts: one academic with experience in nursing management and work environments and two experts conducting research on AI in nursing. Following expert feedback, a pilot study was conducted with three nurses who shared characteristics similar to those of the study sample. Following the pilot study and based on participant feedback, no major modifications were made to the interview form. However, refinements were implemented, particularly in questions about AI use in the nursing work environment. Instead of using a general reference to AI, more specific terminology, such as AI‐supported technologies and tools, was incorporated to enhance clarity and contextual relevance. Based on the pilot interviews, the semi‐structured interview form was finalized (Table 2). The findings from the three pilot interviews were not included in the primary study.

**TABLE 2:** Semi‐structured questions.

Introduction	When you hear the term “artificial intelligence,” what comes to your mind? Could you elaborate? When you hear the term “AI‐supported technologies,” what comes to your mind? Could you elaborate further?
Transition	Are there any AI‐supported technologies that you currently use or know of in the field of nursing? If so, could you provide examples?
Key Questions	What are the areas in which AI‐supported technologies are utilized in the field of nursing? Could you provide examples to explain? What are the areas in which AI‐supported technologies are utilized in the delivery of nursing services? Could you provide examples to explain? What could be the positive or negative impacts of using AI‐supported technologies on the nursing work environment? Could you share your views and experiences? Could you share any positive or negative experiences you have encountered while using AI‐supported technologies in nursing applications? What could be the advantages and disadvantages of using AI‐supported technologies for nurse managers? Could you share your experiences and observations? What factors facilitate or hinder nurses in utilizing AI‐supported technologies in their work environment? Could you share your experiences and observations? What are your views and experiences regarding the potential future impacts of AI‐supported technologies on the nursing work environment? In your opinion, what measures should be taken to enable nurses to use AI‐supported technologies effectively? Do you have any recommendations?
Follow‐up questions	Could you tell me more about that? Could you give me an example? Are there any other examples?
Closing	Is there anything else you would like to add?

Data were collected using the semi‐structured interview form through individual in‐depth interviews. Nurse managers who voluntarily agreed to participate and provided informed consent were contacted and interviews were scheduled at times convenient for the participants. The interviews were conducted online. Before the interviews, participants were informed about the research and notified that the interviews would be recorded with their consent. The interviews lasted approximately 45 minutes on average. Data collection continued until data saturation was achieved. Audio recordings of the interviews were transcribed and reported.

### 3.6 Data Analysis

Content analysis was used for qualitative data analysis (Polit and Beck 2012). To maintain participant confidentiality, nurses were coded as P1, P2, P3, etc. The interviews were recorded, and transcriptions were conducted by the researchers. An inductive approach was used for coding, allowing themes to emerge directly from the data rather than being predetermined (Vears and Gillam 2022). The coding process began with open coding, where two researchers independently performed line‐by‐line coding to identify meaningful units. Using MAXQDA 24, initial codes were systematically assigned to text segments, participants' quotations were included under the codes, and related codes were grouped to form preliminary categories. The software facilitated the organization and visualization of these codes, enabling researchers to iteratively refine and merge them into broader subthemes and overarching themes. These codes were then categorized through constant comparison. Any discrepancies were resolved through joint discussions and consensus among the researchers (Polit and Beck 2012).

### 3.7 Rigor and Trustworthiness

The Lincoln and Guba (1986) criteria (credibility, dependability, confirmability, and transferability) were used to ensure data rigor and trustworthiness. All participants gave consent to participate in the study. To achieve consistency, interviews were conducted by only one of the researchers, who summarized each interview session for participants before asking for additional feedback and responses. To ensure consistency and validity of the data, the main themes and subthemes were identified separately by each researcher, after which consensus was reached through discussion. Moreover, recognizing the potential influence of the research team's expertise in nursing and AI on participant responses, several measures were taken to mitigate bias. The research team adhered to qualitative research reflexivity principles (Braun and Clarke 2019), maintaining neutrality throughout the interviews. Additionally, member checking was conducted, enabling participants to review and refine their statements for accuracy. A semi‐structured interview approach was used to maintain neutrality, and researchers adhered to reflexivity principles to minimize preconceptions affecting data interpretation (Braun and Clarke 2019). Anonymization was applied throughout the coding process to ensure that responses were analyzed objectively (Vears and Gillam 2022). Furthermore, the maximum variation in sampling enhanced transferability and data stability, ensuring various perspectives across different managerial levels. Allocating sufficient time for each interview further strengthened data credibility, allowing participants to fully articulate their experiences (Lincoln and Guba 1986). The COREQ checklist was followed to ensure transparency and rigor in reporting (Tong et al. 2007).

### 3.8 Ethical Considerations

Ethics committee approval was obtained from the Koç University Committee on Human Research (Approval number: 2024.225.IRB3.091). The purpose of the study was explained to all participants before obtaining written and verbal consent. The researchers introduced themselves, explained the purpose of the study, and outlined the data collection method. Written informed consent was obtained from participants who agreed to take part in the study and consented to audio recording. The informed consent form emphasized that participation in the study was entirely voluntary. The time and place of the interviews were arranged according to the participants’ preferences. Participants were informed of their right to withdraw from the study at any time, either before or during their participation. Assurance of data was provided to participants at all stages of the research. Additionally, participants were encouraged to contact the researchers if they had any questions or concerns.

## 4 Results

### 4.1 Participants

This qualitative study involved 22 participants, with a mean age of 29.64 ± 4.45 years. Participants were categorized into two age groups: those aged 29 or older represented the majority at 55%, while 45% were younger than 29. The sample was predominantly female (77.27%). Regarding educational qualifications, most participants hold a bachelor's degree (68.18%). Regarding workplace distribution, 59% of participants were employed in university hospitals. Among professional roles, the first‐line nurse manager constituted the largest group (40.90%), followed by executive/top‐level nurse manager (31.83%) and midlevel nurse manager (27.27%). The mean managerial experience was 7.78 ± 4.73 years (Table 1).

In this study, which explores the perspectives and experiences of nurse managers regarding the impact of AI on nursing work environments and management processes, four main themes were identified: Applications of AI in nursing practice, Reflections of AI on the nursing environment, Barriers to AI use in nursing, and Strategies and drivers for AI integration in nursing practice. Additionally, 12 subthemes were derived from these main themes (Table 3).

**TABLE 3:** Perspectives and experiences of nurse managers on the impact of AI on nursing work environments and managerial processes: Themes and subthemes.

Themes	Subthemes
AI in nursing practice	Nursing practice and patient care Nursing services management
Reflections of AI on the nursing environment	Benefits for nursing practice Patient care quality and safety Benefits for nurse managers Professional development opportunities
Barriers to AI use	Organizational factors Workforce and environmental challenges Nurse attitudes toward AI
Strategies and drivers for AI integration in nursing practice	Organizational readiness Organizational support and culture Empowering nurse managers through AI leadership

#### 4.1.1 Main Theme 1: AI in Nursing Practice

Content analysis of interviews with nurse managers revealed a multifaceted perspective on the integration of AI in nursing practice. Participants' narratives highlighted two main areas where AI has significantly impacted their work: nursing practice and patient care and nursing services management.

#### 4.1.2 Nursing Practice and Patient Care

Participants consistently emphasized how AI has enhanced the quality of patient care by supporting evidence‐based practices and facilitating critical aspects of clinical decision‐making. Another common theme was using AI in care planning and medication administration. Several participants noted that AI‐assisted diagnosis and patient classification tools have streamlined their workflows, reducing the risk of errors. The integration of AI into patient record management also emerged as a significant benefit. Participants mentioned how AI has simplified the documentation process and ensured that critical information is readily accessible when needed.

For example, in our hospital, we use some AI‐powered decision support systems. These systems analyze patient information, such as age, gender, and current health status, to generate specific risk scores. They are particularly helpful in predicting risks like infection, falls, or pressure injuries. (P. 13)

#### 4.1.3 Nursing Services Management

AI's role in nursing services management was described as transformative, particularly in areas such as workforce planning and resource allocation. Resource allocation was another area where participants highlighted the utility of AI. The ability to analyze data and predict resource demands has allowed managers to avoid shortages and optimize the distribution of medical supplies and personnel. Participants also reflected on AI's role in performance management and crisis response. In addition, AI was seen as a critical ally in fostering professional growth.

….For instance, I sometimes use these systems to monitor and keep the inventory updated, helping me manage the resources of the workplace more effectively. (P. 18)

#### 4.1.4 Main Theme 2: Reflections of AI on the Nursing Environment

As a result of the content analysis, the following subthemes were identified: benefits for nursing practice, patient care quality and safety, advantages for nurse managers, and professional development opportunities.

#### 4.1.5 Benefits for Nursing Practice

The integration of AI into nursing practice has been perceived as a transformative tool that enhances various aspects of professional workflows. Participants highlighted that AI supports analytical decision‐making by providing data‐driven insights, enabling more evidence‐based care planning. This not only improves efficiency but also offers significant practicality in daily tasks, contributing to time‐saving and reduced workload. Moreover, AI's capability to enhance staff safety, particularly in high‐risk environments, and its role in data documentation and storage were underscored as critical enablers for streamlining task management. Some participants specifically noted the potential of AI in accident reduction, which aligns with organizational goals for risk mitigation.

As we know, workload and the high number of patients are among the biggest challenges for nurses. I think AI helps reduce our workload because it can handle some routine tasks. For example, AI technologies make tasks like patient monitoring, medication reminders, and organizing patient records more efficient and, honestly, more evidence‐based. (P. 20)

#### 4.1.6 Patient Care Quality and Safety

AI's role in improving patient care quality and safety emerged as a prominent subtheme. Participants emphasized that AI‐driven early warning systems significantly aid in fall prevention and the management of pressure ulcers, ensuring proactive intervention. Furthermore, AI's contribution to preventing adverse events, such as medication errors, was viewed as essential for advancing cost‐effective care. Several participants attributed AI to a noticeable reduction in patient mortality rates, reinforcing its importance in enhancing clinical outcomes.

Additionally, in our institution, there are AI‐integrated medication management systems designed to enhance patient safety. These systems allow us to scan the patient's wristband to ensure they receive their specific medications, with features like dose adjustment. It's a fully patient‐protective system. (P. 15)

#### 4.1.7 Benefits for Nurse Managers

For nurse managers, AI was seen as a valuable asset for patient flow management and workforce planning, allowing more precise resource optimization. The participants reflected AI's ability to minimize waste and increase efficiency, particularly in inventory tracking and team collaboration. The ability to dynamically respond to operational demands with AI's support was identified as a key factor in achieving improved managerial outcomes.

I think AI offers many advantages for nurse managers. It provides great convenience, especially in workforce management, resource allocation, and patient monitoring. We can track patient flow with early warning systems, which helps us use our resources more efficiently. (P. 9)

#### 4.1.8 Professional Development Opportunities

Participants mentioned how AI creates opportunities for professional development, fostering new career trajectories such as nursing engineers, specialists in robotics in nursing, and nursing informatics experts. These emerging roles were seen as empowering, contributing to an enhanced professional image for nurses. Additionally, participants noted the potential influence of AI on healthcare policy, positioning nurses as critical stakeholders in shaping the future of care. Of particular interest was the role of Generation Z nurses, who were identified as agents of transformation and were adept at leveraging AI to drive innovation and change.

By improving patient care outcomes through artificial intelligence, we can strengthen our professional image and enable our profession to play a more active role in healthcare policies. As nurses, we can demonstrate the economic and physiological benefits of AI‐supported care, which could even drive changes in health policy. It has the potential to make a significant contribution to reducing patient costs. (P. 2)

#### 4.1.9 Main Theme 3: Barriers to AI Use

Content analysis revealed several barriers to adopting AI in nursing, categorized under three subthemes: organizational factors, workforce and environmental challenges, and nurse attitudes toward AI.

#### 4.1.10 Organizational Factors

Organizational factors were frequently emphasized as significant obstacles. Participants described how inadequate physical conditions, such as outdated facilities and infrastructure, hinder the adoption of advanced AI technologies. Technology and equipment deficiencies further exacerbated these challenges, with many nurses reporting that the lack of necessary tools and devices prevented them from fully utilizing AI. Financial constraints were another common concern, as limited budgets restricted investments in AI systems and training programs. Moreover, participants highlighted the lack of institutional support for training and technical resources, which they perceived as critical for successfully implementing AI in their workflows.

… As I mentioned, we don't even have the necessary infrastructure or computers. Additionally, since investments in such technologies are often limited in public hospitals, and there is a lack of training and awareness in this area, the widespread implementation of these systems can take time. (P. 4)

#### 4.1.11 Workforce and Environmental Challenges

Participants reported that heavy workloads and high patient ratios left little time or energy for learning and incorporating new technologies. Staff turnover was another significant issue, as frequent changes in team composition disrupted continuity and slowed down the adoption process. Generational differences within the workforce, particularly between more experienced nurses and newer graduates, were noted to create disparities in AI readiness. While some participants appreciated the enthusiasm of new graduates, they also expressed concerns about inadequate skill levels, which posed a challenge to the team‐wide implementation of AI tools.

Our turnover rate is very high, and we mostly work with young and newly graduated nurses. Honestly, as a manager, if I were to go to upper management and request a budget for an AI‐supported system, they might say, “First, focus on keeping up with patient care; that is a secondary concern.” I don't think they would allocate additional resources for AI, as there isn't much of a vision for such investments anyway. (P. 22)

#### 4.1.12 Nurse Attitudes Toward AI

Managers expressed trust regarding the reliability and accuracy of AI systems, alongside concerns about data privacy, security, and ethics. Fear of increased workload was another common sentiment, with some nurses perceiving AI as an additional responsibility rather than a supportive tool. Resistance to change, particularly among older generations, was described as a significant cultural barrier. Finally, knowledge gaps about AI technologies and their applications further limited acceptance, highlighting the need for targeted education and awareness initiatives.

In some areas, AI can create doubts, especially among more experienced nurses who tend to be more resistant. They might wonder, “Will my workload increase? What if the system evaluates something incorrectly or makes an error? This system seems slow—will it add to my workload?” Concerns also arise about data privacy, such as, “All my patient's information is stored here, what if it gets exposed? What about data confidentiality?” These doubts can lead to hesitation in adopting AI systems. (P. 15)

#### 4.1.13 Main Theme 4: Strategies and Drivers for AI Integration in Nursing Practice

Participants identified several strategies and drivers that facilitate the integration of AI into nursing practice, categorized into three subthemes: organizational readiness, organizational support and culture, and empowering nurse managers through AI leadership.

#### 4.1.14 Organizational Readiness

Organizational readiness emerged as a fundamental factor influencing AI integration. Participants consistently emphasized the importance of designing user‐friendly systems that align with the fast‐paced and complex nature of nursing workflows. Additionally, the availability of advanced equipment and reliable technological infrastructure was highlighted as critical for enabling smooth adoption. A dedicated budget for AI implementation was also identified as an essential driver, ensuring the allocation of resources for sustained technological advancement.

The most important facilitating factor is the design of AI systems to be user‐friendly. Having a simple and intuitive user interface is crucial for beginning to learn and adopt technology. Additionally, investments by institutions in nursing, the nursing workforce, and technology can significantly ease the adaptation process to AI. (P. 9)

#### 4.1.15 Organizational Support and Culture

This is recognized as pivotal in facilitating AI adoption. In particular, including nurses in decision‐making processes was noted to enhance their sense of ownership and reduce resistance to change. The establishment of technical support teams to address operational challenges and provide on‐demand assistance was frequently mentioned as a necessary strategy. Furthermore, participants highlighted the role of multidisciplinary collaboration, involving nurses, IT professionals, and other stakeholders, in fostering shared ownership and streamlined implementation. Opportunities for training and professional development, such as participation in workshops, conferences, and scientific events, were reported as critical for equipping nurses with the skills and confidence to effectively use AI technologies.

To help nurses use AI‐based technology more comfortably, hands‐on training sessions should be conducted with a multidisciplinary team, including nurses, physicians, and IT staff. Additionally, personnel supporting the technological infrastructure should always be accessible. Lastly, to ensure staff truly see the benefits of AI applications, pilot projects could be implemented to allow them to experience these processes firsthand. (P. 18)

#### 4.1.16 Empowering Nurse Managers Through AI Leadership

Leadership was identified as a key enabler for driving change. Participants emphasized the importance of transformational leadership, where nurse managers serve as role models by demonstrating openness to innovation and fostering a supportive environment. Effective communication was highlighted as a vital strategy for ensuring that teams clearly understand the benefits and goals of AI integration, thereby enhancing their willingness to embrace new technologies.

Additionally, it's important for managers to be open‐minded about these new technologies and to help nurses adapt to these changes. I believe having visionary leaders who are ready for change and can guide their teams through the transition is a significant factor in making the process easier. (P. 19)

## 5 Discussion

This study aims to understand nurse managers' perceptions and experiences regarding the impact of AI technologies on nursing work environments and management processes. It is structured around four main themes, encompassing the role of AI in the integration into nursing practice, its reflections on work environments, barriers to its use, and strategies and drivers for its integration. This research provides comprehensive insights into the functionality of AI in nursing practices, its contributions to patient safety and care quality, how it transforms nurse managers’ decision‐making and resource management processes, and its potential as a tool for driving change in healthcare policies.

### 5.1 The Contributions of AI in Nursing Practices and Work Environment

Our study aligns significantly with previous research regarding the contributions of AI to nursing practices and patient care. Specifically, participants highlighted the convenience provided by AI in areas such as clinical decision support systems, patient record management, and medication management, supporting Yelne et al.’s (2023) findings that AI is effective in reducing error rates and promoting evidence‐based care processes. A finding of our study is the participants' emphasis on AI's role in reducing nurses’ routine workload, thereby increasing the time spent on direct patient care. Similarly, studies in the literature report that AI‐supported technologies and applications reduce nurses' workload, thereby increasing the time allocated for patient care (Almagharbeh 2024; Ventura‐Silva et al. 2024). AI‐supported technologies and applications have the potential to contribute to a more balanced workload and foster a positive work environment for nurses (Almagharbeh 2024). Additionally, some participants underscored AI's role in enhancing patient safety. Early‐warning systems used for fall prevention and medication error reduction were identified as critical tools for improving patient outcomes. Literature also emphasizes the effectiveness of such systems in reducing mortality rates (Shaw et al. 2023; O'Connor et al. 2023). Shaw et al. (2023) demonstrated that AI‐driven early warning systems reduced in‐hospital mortality by 25% by identifying patient deterioration earlier, allowing for timely interventions. Similarly, Nashwan et al. (2025) highlighted that AI‐supported clinical decision tools enhance patient monitoring, particularly in high‐risk settings, reducing preventable adverse events. These findings suggest that the contributions of AI to patient safety facilitate the adoption of proactive approaches in nursing practices, sustainably enhancing care quality. In addition, it can be said that AI practices in health and nursing contribute to the creation and maintenance of a healthy nursing work environment.

### 5.2 Contributions to Nursing Management and Health Policies

The impact of AI on nursing management processes was prominently demonstrated in this study. Participants reported that AI facilitates decision‐making for managers in areas such as resource management, workforce planning, and performance evaluation. Specifically, the benefits provided by AI‐supported predictive tools for crisis management and organizational resource optimization can contribute to the development of more sustainable management strategies in healthcare institutions (Almagharbeh et al. 2025; Ventura‐Silva et al. 2024). Ventura‐Silva et al. (2024) found that AI‐driven predictive analytics significantly improved hospital resource allocation, reducing patient wait times by 30% and optimizing staff scheduling, which aligns with our findings on AI's role in workforce management. Their study also highlighted that AI‐based forecasting models enhanced emergency preparedness by identifying high‐risk periods for patient surges, allowing for proactive adjustments in staffing and resource distribution (Ventura‐Silva et al. 2024). In their systematic review, Gonzalez‐Garcia et al. (2024) found that AI has been progressively integrated into nursing management, demonstrating significant benefits in operational efficiency. The key findings indicate that AI technologies facilitate better resource management, risk assessment, and decision‐making, which aligns with the results of our study. Furthermore, our study highlights that AI can enable nurses to take on more active roles in health policies. Participants emphasized the economic benefits of AI‐supported care, suggesting that nurses could play a more effective role in shaping health policies. These findings indicate that nurses have the potential to transcend their traditional clinical roles and establish themselves as significant contributors to strategic decision‐making processes. Nashwan et al. (2025) further support this argument, suggesting that nursing informatics and AI integration in healthcare policy development can enhance evidence‐based decision‐making and resource allocation. In addition, AI may contribute to the development of new professional roles for nurses in the future.

### 5.3 Barriers and Recommendations

Nurse managers in this study identified organizational factors as one of the most significant barriers to the integration of AI into nursing practices. Participants frequently cited issues such as infrastructure deficiencies, budget constraints, and limited training opportunities. These findings align with Linnen et al. (2019) and Ventura‐Silva et al. (2024) research, which emphasizes the need for increased infrastructure investments and financial resources for the effective utilization of AI in healthcare services. Additionally, participants highlighted workforce‐related challenges, such as generational differences and high nurse turnover rates, which are consistent with the literature on the cultural and structural barriers to AI adaptation (Lora and Foran 2024; Petersson et al. 2022). Our study also underscored nurses' lack of trust in AI, concerns about data security, and fears of increased workload. Almagharbeh (2024), in their study, reported findings consistent with our results, highlighting that nurses continue to face persistent challenges, including insufficient training, concerns regarding data privacy, and technical difficulties. To address these issues, robust data protection frameworks, the development of nursing‐specific ethical guidelines, and transparent policies must be implemented to ensure that AI systems comply with high confidentiality and security standards (Mohammed et al. 2025). In addition, Kotp et al. (2025) emphasize that nurse leaders play a crucial role in overcoming resistance to AI adoption by fostering a culture of digital innovation and ensuring staff are adequately trained in AI‐related competencies. This finding highlights the need for strategic leadership in AI integration to ensure a smoother transition within nursing environments.

Furthermore, our findings indicate that age and experience influence nurses’ and nurse managers’ attitudes toward AI adoption. Resistance was more evident among older professionals, who viewed AI as disruptive and expressed concerns about reliability and ethical implications (Chang et al. 2022). In contrast, younger nurses and managers, particularly those with prior exposure to digital health technologies, demonstrated greater confidence in AI's potential to enhance efficiency (Gonzalez‐Garcia et al. 2024). To address this resistance stemming from age, experience, and other influencing factors, our study participants additionally emphasized the importance of educational programs, awareness‐raising initiatives, and user‐friendly systems to position AI as a supportive tool. Participants’ suggestions regarding the significance of user‐friendly system designs and interdisciplinary collaboration align with the solution strategies outlined by Rony et al. (2024).

### 5.4 Limitations of the Study

The findings of this study are subject to certain limitations, as they are built upon data derived from qualitative interviews. First, the sample group, consisting of a limited number of nurse managers, restricts the generalizability of the findings. The fact that participants were selected solely from a specific geographic region or healthcare system may hinder the assessment of AI's impact across diverse healthcare systems and cultural contexts. Second, the qualitative research method, relying on participants' personal experiences and perceptions, may increase the subjectivity of the findings. Specifically, variations in participants' knowledge levels and personal attitudes toward AI technologies could influence the diversity of insights obtained during the interviews. Additionally, while the study includes relatively young nurse managers, the predominance of younger managers may limit the generalizability of the findings to more senior nursing leadership.

## 6 Conclusion

This study comprehensively reveals both the opportunities and challenges associated with the integration of AI technologies into the nursing work environment, nursing practices, and management processes. The findings suggest that AI has the potential to significantly accelerate the transformation of the nursing profession through its contributions to critical areas such as patient safety, care quality, and resource management. Applications such as clinical decision support systems and patient safety tools not only optimize care processes but also reduce nurses’ workload, thereby promoting patient‐centered care. However, organizational factors, infrastructure deficiencies, budget constraints, nurses' attitudes, data security concerns, and cultural barriers to technology adoption were identified as key obstacles to the effective implementation of AI in nursing. On the other hand, organizational readiness, organizational support and culture, and empowering nurse managers through AI leadership were identified as the main drivers for AI applications in the nursing work environment.

## 7 IMPLICATIONS FOR NURSING PRACTICE AND NURSING POLICY

The findings indicate that AI can serve as a strategic tool not only in nurses' clinical practices but also in shaping nursing leadership and health policies in the nursing work environment. To enhance the effectiveness of these technologies, it is essential to develop user‐friendly system designs, raise awareness among nurses about technology, and implement comprehensive training programs that build trust. Nursing leadership should adopt a comprehensive training model to prepare for AI integration, incorporating in‐service training programs, nurse–manager development courses, and continuous professional education programs. Continuing education programs should include seminars, workshops, and training activities designed to enhance nurse managers’ knowledge and skills in AI‐driven decision‐making, digital transformation strategies, and ethical principles. These programs should equip nurse leaders with the necessary competencies to navigate AI adoption effectively. Additionally, training on the use of AI in nursing work environments and healthcare services should be integrated into undergraduate nursing curricula to ensure that future nurse leaders develop digital health literacy and readiness for emerging technologies. AI competency frameworks should be established to systematically assess individual and institutional AI readiness and guide professional development to ensure that AI training remains measurable and sustainable. Furthermore, establishing AI‐supported technology development and implementation offices within healthcare organizations can enhance structured and ethical AI adoption by actively involving nurses in the process. Policymakers should develop regulatory guidelines that promote training incentives, resource allocation, and data security, ensuring that AI‐driven nursing practices align with professional and ethical standards. By implementing structured and equitable AI integration strategies, nursing leadership can facilitate a seamless transition toward AI adoption, ultimately improving patient care and operational efficiency.

Future research should focus on large‐scale studies to evaluate the long‐term effects of AI and analyze how these technologies can transform health and nursing policies. Specifically, studies examining AI's impact on nursing leadership and decision‐making processes could further enrich the knowledge base in this field. Finally, future research not only examines the effects of the use of AI‐related applications in the field of nursing and health on nursing, patient, and organizational outcomes but also recommends conducting studies to determine the effects of AI in shaping and transforming the conceptual and professional development of nursing.

## Author Contributions

Study design: NG and SS. Data collection: SS. Data analysis: NG and SS. Study supervision: NG. Manuscript writing: NG and SS. Critical revisions for important intellectual content: NG and SS.

## Ethics Statement

The study was approved by the Koç University Committee on Human Research (Approval number: 2024.225.IRB3.091).

## Conflicts of Interest

The authors declare no conflicts of interest.

## Supporting information




**Supporting File1**: inr70043‐sup‐0001‐SuppMat.docx

## References

[inr70043-bib-0001] Almagharbeh, W. T. 2024. “The Impact of AI‐Based Decision Support Systems on Nursing Workflows in Critical Care Units.” International Nursing Review. 72, no. 2: e13011. 10.1111/inr.13011. 38973347

[inr70043-bib-0002] Almagharbeh, W. T. , H. A. Alfanash , K. A. Alnawafleh , et al. 2025. “Application of Artificial Intelligence in Nursing Practice: A Qualitative Study of Jordanian Nurses' Perspectives.” BMC Nursing 24: 92. 10.1186/s12912-024-02658-6. 39863852 PMC11762109

[inr70043-bib-0003] Alsadaan, N. , B. Salameh , F. A. A. E. Reshia , et al. 2023. “Impact of Nurse Leaders Behaviors on Nursing Staff Performance: A Systematic Review of Literature.” Inquiry: A Journal of Medical Care Organization, Provision, And Financing. 60: 469580231178528. 10.1177/00469580231178528. 37269099 PMC10265372

[inr70043-bib-0004] Booth, R. G. , G. Strudwick , S. McBride , S. O'Connor , and A. L. Solano López . 2021. “How the Nursing Profession Should Adapt for a Digital Future.” The BMJ 373: n1190. 10.1136/bmj.n1190.

[inr70043-bib-0005] Braun, V. , and V. Clarke . 2019. “Reflecting on Reflexive Thematic Analysis.” Qualitative Research in Sport, Exercise and Health 11, no. 4: 589–597. 10.1080/2159676X.2019.1628806.

[inr70043-bib-0006] Chang, C. Y. , H. J. Jen , and W. S. Su . 2022. “Trends in Artificial Intelligence in Nursing: Impacts on Nursing Management.” Journal of Nursing Management 30, no. 8: 3644–3653. 10.1111/jonm.13770. 35970485

[inr70043-bib-0007] Chang, H. Y. , T. L. Huang , M. K. Wong , L. H. Ho , C. N. Wu , and C. I. Teng . 2021. “How Robots Help Nurses Focus on Professional Task Engagement and Reduce Nurses' Turnover Intention.” Journal of Nursing Scholarship 53, no. 2: 237–245. 10.1111/jnu.12629. 33567145

[inr70043-bib-0008] Choudhury, A. , and O. Asan . 2020. “Role of Artificial Intelligence in Patient Safety Outcomes: Systematic Literature Review.” JMIR Medical Informatics 8, no. 7: e18599. 10.2196/18599. 32706688 PMC7414411

[inr70043-bib-0009] Douthit, B. J. , X. Hu , R. L. Richesson , H. Kim , and M. P. Cary . 2020. “How Artificial Intelligence Is Transforming the Future of Nursing.” American Nurse Journal 29, no. 10: 1513–1517.

[inr70043-bib-0010] Frazier, R. M. , H. Carter‐Templeton , T. H. Wyatt , and L. Wu . 2019. “Current Trends in Robotics in Nursing Patents: A Glimpse Into Emerging Innovations.” Computers, Informatics, Nursing 37, no. 6: 290–297. 10.1097/CIN.0000000000000538. 31135470

[inr70043-bib-0011] General Directorate of Health Information Systems . 2021. Health Statistics Yearbook 2021. Ministry of Health, Turkey. Retrieved December 25, 2024, from https://www.saglik.gov.tr/TR,95109/saglik‐istatistikleri‐yilligi‐2021‐yayinlanmistir.html.

[inr70043-bib-0012] Gonzalez‐Garcia, N. A. , S. Pérez‐González , C. Benavides , A. Pinto‐Carral , E. Quiroga‐Sánchez , and P. Marqués‐Sánchez . 2024. “Impact of Artificial Intelligence‐Based Technology on Nurse Management: A Systematic Review.” Journal of Nursing Management 2024, no. 1: 3537964. 40224848 10.1155/2024/3537964 PMC11919197

[inr70043-bib-0013] Hassan, E. A. , and A. M. El‐Ashry . 2024. “Leading With AI in Critical Care Nursing: Challenges, Opportunities, and the Human Factor.” BMC Nursing 23: 752. 10.1186/s12912-024-02363-4. 39402609 PMC11475860

[inr70043-bib-0014] Khatatbeh, H. , A. Pakai , T. Al‐Dwaikat , et al. 2022. “Nurses' Burnout and Quality of Life: A Systematic Review and Critical Analysis of Measures Used.” Nursing Open 9, no. 3: 1564–1574. 10.1002/nop2.936. 33991408 PMC8994939

[inr70043-bib-0015] Kotp, M. H. , H. A. Ismail , H. A. Basyouny , et al. 2025. “Empowering Nurse Leaders: Readiness for AI Integration and the Perceived Benefits of Predictive Analytics.” BMC Nursing 24: 56. 10.1186/s12912-024-02653-x. 39819624 PMC11737245

[inr70043-bib-0016] Lincoln, Y. S. , and E. E. Guba . 1986. “Research, Evaluation, and Policy Analysis: Heuristics for Disciplined Inquiry.” Review of Policy Research 5, no. 3: 546–565.

[inr70043-bib-0017] Linnen, D. T. , P. S. Javed , and J. N. D'Alfonso . 2019. “Ripe for Disruption? Adopting Nurse‐Led Data Science and Artificial Intelligence to Predict and Reduce Hospital‐Acquired Outcomes in the Learning Health System.” Nursing Administration Quarterly 43, no. 3: 246–255. 10.1097/NAQ.0000000000000356. 31162343

[inr70043-bib-0018] Locsin, R. C. 2017. “The Co‐Existence of Technology and Caring in the Theory of Technological Competency as Caring in Nursing.” Journal of Medical Investigation 64, no. 1–2: 160–164. 10.2152/jmi.64.160. 28373615

[inr70043-bib-0019] Lora, L. , and P. Foran . 2024. “Nurses' Perceptions of Artificial Intelligence (AI) Integration Into Practice: An Integrative Review.” Journal of Perioperative Nursing 37, no. 3: e–28.

[inr70043-bib-0020] Mohammed, S. A. A. Q. , Y. M. M. Osman , A. M. Ibrahim , and M. Shaban . 2025. “Ethical and Regulatory Considerations in the Use of AI and Machine Learning in Nursing: A Systematic Review.” International Nursing Review 72, no. 1: e70010. 10.1111/inr.70010. 40045476

[inr70043-bib-0021] Nashwan, A. J. , J. A. Cabrega , M. I. Othman , et al. 2025. “The Evolving Role of Nursing Informatics in the Era of Artificial Intelligence.” International Nursing Review 72, no. 1: e13084. 10.1111/inr.13084. 39794874 PMC11723855

[inr70043-bib-0022] Ng, Z. Q. P. , L. Y. J. Ling , H. S. J. Chew , and Y. Lau . 2022. “The Role of Artificial Intelligence in Enhancing Clinical Nursing Care: A Scoping Review.” Journal of Nursing Management 30, no. 8: 3654–3674. 10.1111/jonm.13425. 34272911

[inr70043-bib-0023] O'Connor, S. , Y. Yan , F. J. Thilo , H. Felzmann , D. Dowding , and J. J. Lee . 2023. “Artificial Intelligence in Nursing and Midwifery: A Systematic Review.” Journal of Clinical Nursing 32, no. 13–14: 2951–2968. 10.1111/jocn.16478. 35908207

[inr70043-bib-0024] Oksanen, A. , N. Savela , R. Latikka , and A. Koivula . 2020. “Trust Toward Robots and Artificial Intelligence: An Experimental Approach to Human‐Technology Interactions Online.” Frontiers in Psychology 11: 568256. 10.3389/fpsyg.2020.568256. 33343447 PMC7744307

[inr70043-bib-0025] Petersson, L. , I. Larsson , J. M. Nygren , et al. 2022. “Challenges to Implementing Artificial Intelligence in Healthcare: A Qualitative Interview Study With Healthcare Leaders in Sweden.” BMC Health Services Research 22, no. 1: 850. 10.1186/s12913-022-08215-8. 35778736 PMC9250210

[inr70043-bib-0026] Polit, D. F. , and C. T. Beck . 2012. Nursing Research: Generating and Assessing Evidence for Nursing Practice (9th ed.). Lippincott Williams & Wilkins.

[inr70043-bib-0027] Ramadan, O. M. E. , M. M. Alruwaili , A. N. Alruwaili , et al. 2024. “Facilitators and Barriers to AI Adoption in Nursing Practice: A Qualitative Study of Registered Nurses' Perspectives.” BMC Nursing 23: 891. 10.1186/s12912-024-02571-y. 39695581 PMC11654280

[inr70043-bib-0028] Robert, N. 2019. “How Artificial Intelligence Is Changing Nursing.” Nursing Management 50, no. 9: 30–39. 10.1097/01.NUMA.0000578988.56622.21. PMC7597764 31425440

[inr70043-bib-0029] Rogers, E. 2003. Diffusion of Innovations. Fifth edition. Free Press.

[inr70043-bib-0030] Roncarolo, F. , A. Boivin , J. L. Denis , et al. 2017. “What Do We Know About the Needs and Challenges of Health Systems? A Scoping Review of the International Literature.” BMC Health Services Research 17: 636. 10.1186/s12913-017-2585-5. 28886736 PMC5591541

[inr70043-bib-0031] Rony, M. K. K. , S. M. Numan , F. T. Johra , et al. 2024. “Perceptions and Attitudes of Nurse Practitioners Toward Artificial Intelligence Adoption in Health Care.” Health Science Reports 7, no. 8: e70006. 10.1002/hsr2.70006. 39175600 PMC11339127

[inr70043-bib-0032] Ross, A. , R. Freeman , K. McGrow , and O. Kagan . 2024. “Implications of Artificial Intelligence for Nurse Managers.” Nursing Management 55, no. 7: 14–23. 10.1097/nmg.0000000000000143. 38951725

[inr70043-bib-0033] Shaw, P. , K. Pachpor , and S. Sankaranarayanan . 2023. “Explainable AI‐Enabled Infant Mortality Prediction Based on Neonatal Sepsis.” Computer Systems Science & Engineering 44, no. 1: 311–325. 10.32604/csse.2023.025281.

[inr70043-bib-0034] Tong, A. , P. Sainsbury , and J. Craig . 2007. “Consolidated Criteria for Reporting Qualitative Research (COREQ): A 32‐Item Checklist for Interviews and Focus Groups.” International Journal for Quality in Health Care 19, no. 6: 349–357. 10.1093/intqhc/mzm042. 17872937

[inr70043-bib-0035] Vears, F. D. , and D. Gillam . 2022. “Inductive Content Analysis: A Guide for Beginning Qualitative Researchers.” Focus on Health Professional Education: A Multi‐Professional Journal 23, no. 1: 111–127. 10.11157/fohpe.v23i1.544.

[inr70043-bib-0036] Ventura‐Silva, J. , M. M. Martins , L. D. L. Trindade , et al. 2024. “Artificial Intelligence in the Organization of Nursing Care: A Scoping Review.” Nursing Reports 14, no. 4: 2733–2745. 10.3390/nursrep14040202. 39449439 PMC11503362

[inr70043-bib-0037] Yelne, S. , M. Chaudhary , K. Dod , A. Sayyad , and R. Sharma . 2023. “Harnessing the Power of AI: A Comprehensive Review of Its Impact and Challenges in Nursing Science and Healthcare.” Cureus 15, no. 11: e49252. 10.7759/cureus.49252. 38143615 PMC10744168

[inr70043-bib-0038] Zhao, Z. , Y. Ma , A. Mushtaq , et al. 2022. “Applications of Robotics, Artificial Intelligence, and Digital Technologies During COVID‐19: A Review.” Disaster Medicine and Public Health Preparedness 16, no. 4: 1634–1644. 10.1017/dmp.2021.9. 33413717 PMC8027549

